# MDM4 overexpression alleviates developmental and hematopoietic defects in Fancg deficient mice

**DOI:** 10.1038/s41375-025-02692-6

**Published:** 2025-07-25

**Authors:** Maeva Loock, Carèle Fédronie, Carine Torset, Lucie Hernandez, Emmanuelle Latour, Samuel Quentin, Nathalie Gachard, Julie Lefrançois, Loïc Maillard, Francina Langa Vives, Véronique Parietti, Marie-Laure Arcangeli, Camille Lobry, Jean Soulier, Olivier Bluteau, Dominique Bluteau

**Affiliations:** 1 CNRS UMR9019, Paris Saclay University, Gustave Roussy Cancer Campus, Villejuif, France; 2 Université Paris Cité, Institut de Recherche Saint-Louis (IRSL), INSERM UMR1342/CNRS EMR8000, Paris, France; 3 Hematology Laboratory, Hopital Saint-Louis, APHP, Paris, France; 4 INSERM U1342, Institut de la Leucémie Paris Saint-Louis, Université Paris-Cité, Paris, France; 5 Hematology laboratory, CHU Limoges, Limoges, France; 6 Mouse Genetics Engineering Center, Pasteur Institute, Paris, France; 7 UMS Saint-Louis (US53/UAR2030), University Paris Cité, INSERM, CNRS, Paris, France; 8 INSERM U1170, Paris Saclay University, Gustave Roussy cancer campus, Villejuif, France; 9 INSERM UMRS 1166 - ICAN, Faculty of Medicine Pitié-Salpétrière, Sorbonne University, Paris, France; 10 Service de Biochimie Endocrinienne et Oncologique, Assistance publique - Hôpitaux de Paris Sorbonne Université Pitié Salpêtrière, Paris, France; 11 EPHE, PSL University, Paris, France

**Keywords:** Haematopoietic stem cells, Cancer models

**To the Editor:**

Fanconi anemia (FA) is the most common inherited cause of bone marrow failure (BMF). Most FA patients experience hematopoietic stem cell (HSC) attrition and cytopenia during childhood. Along with intrinsic chromosomal instability, these conditions favor clonal evolution and frequently lead to the emergence of myelodysplastic neoplasm (MDS) and acute myeloid leukemia (AML) in their teens or early adulthood. To date, 23 Fanconi Anemia (FA) genes have been identified. They encode proteins that cooperate in the FA pathway whose primary function is the repair of DNA interstrand crosslinks (ICLs) [1]. Moreover, FA pathway is also at the crossroads of several biological processes and its impairment results in multiple cellular dysfunctions such as genotoxicity from endogenous ICL agents, aldehydes [2], physiological proliferative stress [3], elevated p53 levels [4], *MYC* oncogene overexpression [5], hypersensitivity to inflammatory cytokines, oxidative stress and a hyperactive TGF beta pathway [6]. These alterations contribute to the loss of hematopoietic stem and progenitor cells (HSPC) that occurs in FA patients. Currently, allogeneic transplantation remains the only curative therapy to restore hematopoiesis and improve patient survival. Recently, we identified a distinct pattern of somatic structural variants and mutations that engage the cells towards MDS and AML in FA. The hallmark of these alterations consists of microhomology-mediated chromosomal rearrangements leading to copy-number alterations. The most characteristic is the chromosome 1q gain, which drives clonal hematopoiesis through the *MDM4* duplication that dampens p53 signaling [7]. Although FA mouse models, including *Fancg*^*−/−*^ mice, partially recapitulate human developmental alterations and hematopoietic defects [8–10], further investigations are required to elucidate the mechanisms predisposing hematopoietic FA cells to leukemic transformation. In this study, we have developed a joint molecular and cellular approach to investigate the “pre-leukemic” state in FA. We generated a *Mdm4* transgenic mouse strain, *Fancg*^*+/+*Tg(RPL23-203515)^ (WTTg), by introducing a wide genomic region surrounding *Mdm4* that includes its proximal regulatory sequences, using a bacterial artificial chromosome (BAC; *RPL23-203E15)* which resulted in the *Mdm4* overexpression (Supplementary Fig. S1A) [7]. Therefore, we crossed *Fancg*^*+/−*^ and *Fancg*^*+/−*Tg(RPL23-203515)^ mice (Supplementary Fig. S1B) to obtain *Fancg*^*−/−*^ (KO), *Fancg*^*−/−*Tg(RPL23-203515)^ (KOTg), *Fancg*^*+/+*Tg(RPL23-203515)^ (WTTg) and *Fancg*^*+/+*^ (WT) progeny. To verify the stability of *Mdm4* expression over time, we measured *Mdm4* RNA expression levels in the progeny of KOTg and WTTg. As evidenced in Supplementary Fig. S1, *Mdm4* overexpression remain stable in several lineages including PBMC (Supplementary Fig. S1C), Lin^−^ SCA1^+^ KIT^+^ (LSK) and progenitor cells (Supplementary Fig. S1D).

We assessed the consequences of alteration of the FA pathway in HSPCs in KO or in KOTg mice at steady state and under Poly-IC (pIC) induced stress.

As previously reported [11], *Fancg*^−/−^ mice exhibited perinatal death (Fig. 1A), growth retardation (Supplementary Fig. S2A) and developmental defects of the skull and the eyes (Fig. 1B, Supplementary Fig. S2B). We also observed a decrease of in vitro hematopoietic clonogenic capacity (Fig. 1C), an increased sensitivity to mitomycin C (MMC) (Fig. 1D) and a defect in engraftment capacity in comparison to WT cells (Fig. 1E). Interestingly, constitutive *Mdm4* overexpression in KOTg mice rescued the survival, reduced developmental phenotypes and alleviated hematopoietic defects observed in KO mice (Fig. 1A–F). However, after over 300 days of follow-up neither WTTg nor KOTg mice developed tumor transformation. This result confirms that as in human FA, *Mdm4* overexpression alone is not sufficient to induce cellular transformation.

**Fig. 1:**
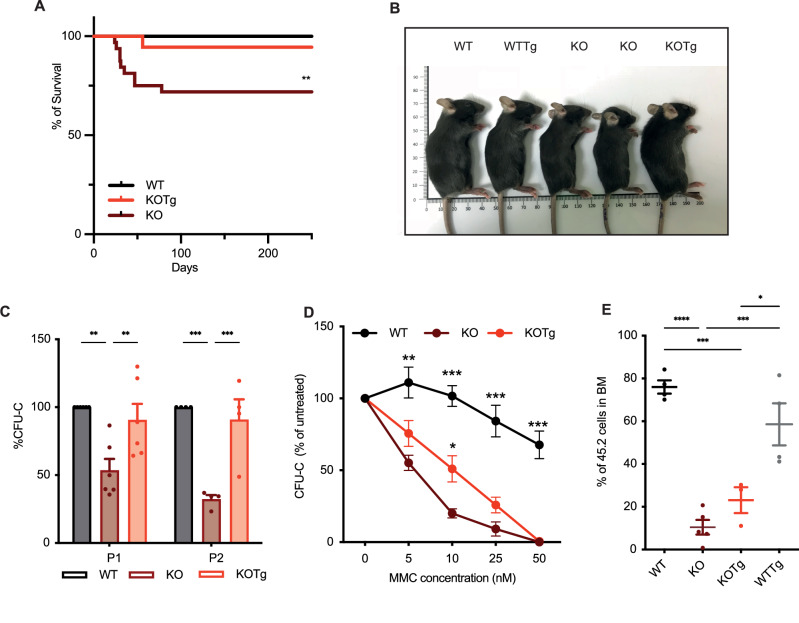
*Mdm4* overexpression partially rescued developmental and hematopoietic defects of KO mice. **A** Survival of KO mice compared to WT and KOTg mice. *P* = 0.007 for KO compared with WT by the log-rank test for trend. **B** Representative image of 2 months old WT, WTTg, KO and KOTg mice. **C** Percent of CFU-C compared to WT. Colonies (primary CFU-C) of 3000 BM Lin– cells were quantified after 7 days in methylcellulose (P1) (*n* = 6); Cells recovered from primary CFU-C plates were seeded in new CFU-C medium to perform serial CFU-C assay. Total cells (1%) of equivalent primary plates were seeded in triplicate. Secondary CFU-C was quantified 7 days later (P2), (*n* = 4); **D** Impaired clonogenic capacity of WT (*n* = 4), KOTg (*n* = 5) and KO (*n* = 5) cells over 10-day treatment with MMC. Data represent mean ± SEM of independent experiments each of which was plated in triplicate cultures isolated from the BM of 8–12 weeks-old syngeneic mice, multiple unpaired t-test was used to determine *p*-values. **E** Percent of CD45.2 cells in recipient mice, 16 weeks after transplant. Experimental design: WT, KO, KOTg or WTTg BMCs (CD45.2) and isogenic WT (CD45.1) cells were co-transplanted into lethally irradiated recipient mice (45.1). Each symbol represents an individual recipient animal; large horizontal bars represent the mean of each group. Error bars represent mean ± SEM, two-ways ANOVA was used to determine *p* values.

To finely depict the consequences of *Mdm4* overexpression in the hematopoietic compartment and to understand the setup of the pre leukemic state, we performed RNA-seq analysis on highly purified LSK cells from KO, WT and KOTg individual mice at steady state and 48 h post pIC stress (5 mg/Kg). At steady state, only a limited number of differentially expressed genes were detected in KO LSK cells compared to WT cells, suggesting that only subtle perturbations are tolerable for LSK cells with *Fancg* deletion to survive. Surprisingly, no major transcriptomic differences were observed in KO LSK cells post pIC compared to WT. However, pathway enrichment analysis highlighted significant similarities between KO LSK and WT LSK post pIC stress (Fig. 2A). Using GSEA analysis and in accordance with data obtained in FA patient cells [5], we confirmed that the expression of several key genes involved in HSC stemness was perturbed in KO LSK subsets with an enrichment of TP53 and MYC pathways compared to WT (Fig. 2A, Supplementary Fig. S3A). Notably, except for cell cycle pathway signature, all these enrichment profiles were reverted upon Mdm4 overexpression in LSK KO cells (Fig. 2A, Supplementary Fig. S3A).

**Fig. 2:**
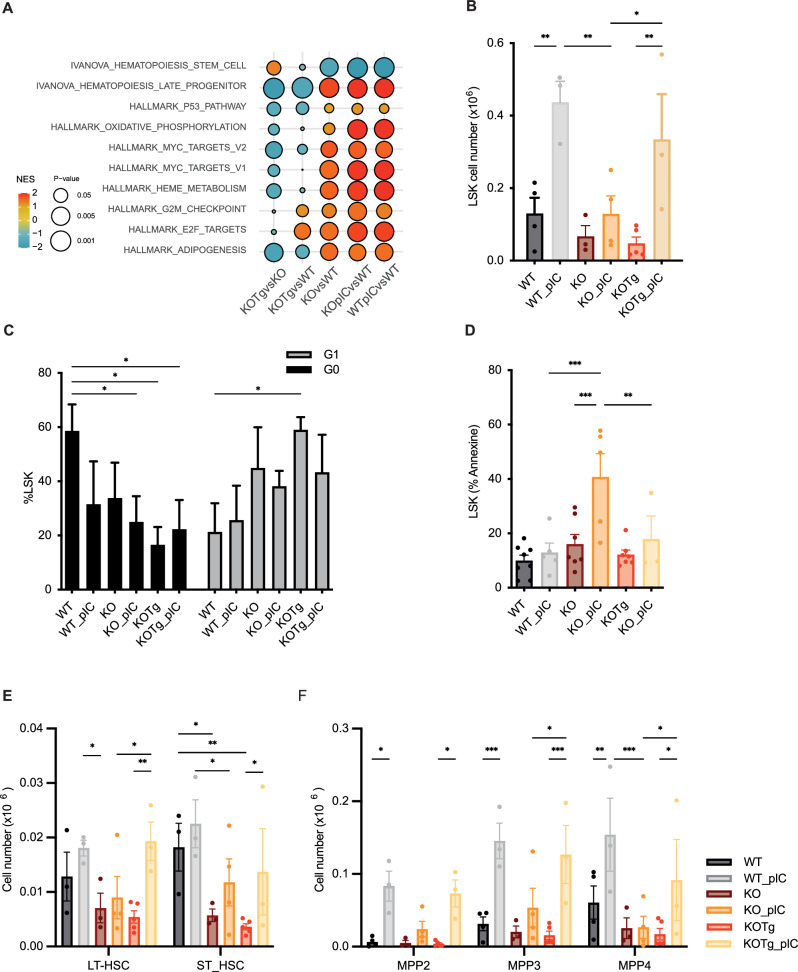
*Mdm4* overexpression decreases apoptosis and restores cells number and inflammatory response under stress of KO LSK cells. **A** GSEA analysis representation from LSK cells, RNAseq analysis. **B** Total number of BM cells in 8–12-weeks old WT (*n* = 4), WT_pIC (*n* = 3), KOTg (*n* = 5), KOTg_pIC (*n* = 3), KO (*n* = 3) and KO_pIC (*n* = 4) mice (mean ± SEM). **C** Cell cycle analysis of LSK cells in 8-12-weeks old WT (*n* = 4), WT_pIC (*n* = 3), KOTg (*n* = 3), KOTg_pIC (*n* = 3), KO (*n* = 4) and KO_pIC(*n* = 4) mice. Error bars represent mean ± SEM, two-ways ANOVA was used to determine p values (*p  <  0.05). **D** Apoptosis analysis of LSK cells in 8–12-weeks old WT (*n* = 9), WT_pIC (*n* = 8), KOTg (*n* = 7), KOTg_pIC (*n* = 3), KO (*n* = 7) and KO_pIC (*n* = 5) mice. Error bars represent mean ± SEM, two-ways ANOVA was used to determine p values (*p  <  0.05). **E** Total number of BM LT-HSC (Lin−Sca-1+c-Kit+CD48−CD150+), ST-HSC (c-Kit+Lin−Sca-1+ CD135− CD34+), or **F** multipotent progenitor (MPP2, Lin−Sca-1+c-Kit+ CD135- CD48+ CD150+), (MPP3, Lin−Sca-1+c-Kit+ CD135- CD48+ CD150-), (MPP4, Lin−Sca-1+c-Kit+ CD135+). Error bars represent mean ± SEM, two-ways ANOVA was used to determine p values (*p 0.05).

Alongside this LSK transcriptomic analysis, we performed an exhaustive cellular study including quantification of apoptosis and cell cycle analysis, with or without stress (Fig. 2B–F). Interestingly, KO mice displayed a reduced LSK population in bone marrow (BM) at steady state and post pIC stress (Fig. 2B), a cell cycle alteration characterized by G1 phase blockade (Fig. 2C) and an increase in apoptosis (Fig. 2D) in comparison to WT cells. The overexpression of Mdm4 rescued the response of KO LSK cells to pIC inflammatory stress, with a number of KOTg LSK cells comparable to the one of WT LSK. However, this overexpression was not sufficient to rescue the number of KO LSK cells in steady state BM (Fig. 2B). The LSK compartment is enriched in HSPC-population but can be further divided into long-term hematopoietic stem cells (LT-HSC), short-term HSC (ST-HSC) or multipotent progenitors (MPP) 2, MPP3 and MPP4 [12] (gating strategy Supplementary Fig. S3B). We showed that WT LSK populations exhibit an inflammatory cellular response post pIC characterized by an increased number of total LSK (Fig. 2B) corresponding to an increased number of cells in MPP2, 3 and 4 compartments (Fig. 2E, F). In post-inflammatory stress, we did not observe a significant increase of KO LSK cells (Fig. 2B), but on contrary a huge increase of cell apoptosis (Fig. 2D) and an attenuated inflammatory cellular response in all LSK subsets (Fig. 2E, F). In addition, the strong increase in apoptosis KO LSK cells (Fig. 2D) was restricted to the MPP3, MPP4 and ST-HSC populations (Supplementary Fig. S3C, D). These results could explain the origin of BM failure observed in FA patients; this could be due to the increase in apoptosis of multipotent progenitor’s cells. The specific effect of *Mdm4* overexpression is the decrease of LSK apoptosis (especially in ST-HSC, MPP3 and MPP4 compartment, Fig. 2B; Supplementary Figs. S4A, S5A) but, in accordance with RNA-seq data, is not sufficient to rescue cell cycle arrest in G1 phase compared to KO mice (Fig. 2C; Supplementary Figs. S4B, S5B). Additionally, we assessed γH2AX and 53BP1 foci in HSPCs cultured in vitro, with or without MMC, using cells derived from WT, KO and KOTg mice (Supplementary Fig. S5C, D). Altogether, these results suggest that KOTg cells are able to survive by tolerating damaged DNA.

In conclusion, we developed an original transgenic mouse model able to determine the impact of *Mdm4* overexpression in the rescue of FA developmental, cellular, and molecular phenotype that are observed in human. This model is designed to allow the innate regulation of *Mdm4* since we included its natural promoter and proximal regulating sequences. Crossing with *Fancg*^−/−^ mice model rescued FA developmental defects but also alleviate cellular and molecular phenotypes observed in these mice. We have clearly shown that overexpression of *Mdm4* inhibits apoptosis in HSPC FA cells under stress, enabling them to survive. However, the blockade of cell cycle observed in KO cells is not rescued by *Mdm4* overexpression, suggesting the involvement of other mechanisms. Unlike FA mouse models crossed with *Trp53* knockout mice [13, 14], KOTg mice do not spontaneously develop tumor transformation over time. *TP53* deletion is one of the most common genomic events observed in squamous cell carcinomas from FA patients [15]. However, *TP53* inactivation is not a common mechanism of FA leukemogenesis. Accordingly with our hypothesis and the natural history of oncogenesis in FA, our model accurately recapitulates features of human FA hematopoietic disease evolution with an attenuation of p53 dependent apoptosis allowing cell survival. This confirms that a complete leukemic transformation will depend on additional genomic events. Therefore, this new model could be considered as a preleukemic/pre-oncogenic model that is relevant in context of FA patient’s genomic alterations previously described. The pre-leukemic state in FA requires additional events to facilitate cellular transformation. Indeed, in RNA-Seq analysis, we also have identified other deregulated genes that were not been previously highlighted but that are functionally highly relevant in FA disease. In particular, we identified several genes whose expression is altered in murine FA-deficient hematopoietic cells, supporting the exacerbation of the TP53 pathway alongside the additional chromosome alterations that occur during the leukemic evolution of FA. In this context, the challenge is now to precisely determine how these targets would be involved in leukemic progression, whether they can be used as new markers of FA disease progression as well as prognostic markers of transformation in patients.

## Supplementary information

Supplementary informations

Supplementary Table 1

Supplementary Table 2
