# Supplementary material for: MDM4 overexpression alleviates developmental and hematopoietic defects in Fancg deficient mice
Source: Leukemia. 2025 Jul 25;39(10):2542–6. doi: 10.1038/s41375-025-02692-6 (PMC12463665; doi:10.1038/s41375-025-02692-6)
Supplement: Supplementary file 1 — Supplementary informations [file 41375_2025_2692_MOESM1_ESM.pdf]

Supplementary informations

Animal models

Fancg$^{-/-}$ mice were a kind gift of F. Arwert(21). *Fancg*$^{+/+Tg(RPL23-203515)}$ (WTTg) and *Fancg*$^{-/-Tg(RPL23-203515)}$ (KOTg) mice have been previously described*(11)*. Transgenic mice for the *Mdm4* gene locus (WTTg) to generate the B6J.B6N-Tg (RPL23-203E15). A mouse, thereafter, referred to WTTg, the 190,044 bp Mouse BAC RPL23-203E15 (ordered at Life technologies SAS), that includes the complete *Mdm4* gene along with 22.006 kb 50 and 128.726 kb 30 flanking regions, was micro-injected in the C57BL/6N background. Genotyping primers are listed in Supplemental Table 2. Stable integration of the BAC was visualized in the WTTg line after several generations crossed with the C57BL/6J using aCGH (Agilent, SurePrint G3 Mouse Genome CGH Microarray 1M). *Mdm4* gene overexpression was checked by specific RT-qPCR using the CAAGCCCTCTCTATGACATGC and ATCCTGTGCGAGAGCGAGA primers.

Animal facility

All mice were housed and handled in the pathogen-free animal facility Département d'Expérimentation Animale in accordance with Institutional Animal Care and Use Committee-approved protocols (IRSL, Saint-Louis Hospital). Authorizations for animal experimentation was obtained with the No. 2014-IUH013 and APAFIS#19958-2019032615074625v2, in accordance with French laws.

Analyses of murine models

Hemoglobin, mean corpuscular volume, hematocrit, RBC counts, platelet, and WBC counts were determined using an automated counter (MS9; Melet Schloesing Laboratoires) on blood collected from the retro-orbital plexus in EDTA tubes. BM cells were collected by crushing bones (tibia, femur). For further analysis of nuclear cells, red blood cells were lysed with lysis buffer (150mM NH_4Cl, 1mM $KHCO_3$ and 0.1mM EDTA). To provoke *in vivo* stress of HSCs, mice were injected with either 5 mg kg^{-1} pI:pC intraperitoneally (i.p.) (InvivoGen).

Flow Cytometry (FCM)

Eight to 12-week-old mice were used for cell isolation, and 8- to 12-week-old CD45.1 C57Bl/6-Boy/J wild-type mice were used as recipients for cell transplantation. Cells analyzed by flow cytometry were antibody-stained in PBS 1× supplemented with 2% FBS for 30 minutes at 4°C with washing prior to FACS analysis. Whole BM cells from mice were collected, subjected to RBC lysis, and stained for FACS analysis $5x10^6$ nuclear cells of BM were stained for FCM analysis using FACS Fortessa (BD Biosciences). The antibodies used for flow cytometry were purchased from BD Bioscience (Le Pont de Claix, France), eBioscience (Thermofisher, Villebon-sur-Yvette, France), Biolegend (AmsterdamThe Netherlands) or Sony Biotechnology (San Jose, CA, USA) and are available in the Supplemental Table 1. The flow cytometry results were analyzed using FlowJo™ v10.9 Software (BD Life Sciences).

RNA-seq study

Mature BM cells were depleted using the direct lineage cell depletion kit (Miltenyi Biotec, Paris France) following manufacturer instructions. The remaining Lin^{neg} cells were stained with Pacific Blue™ Mouse Lineage Antibody Cocktail, anti-CD117PercpCy5.5, ant-Sca-1 PECy7. LSK populations were purified by cell sorting using a FACS-ARIAIII (BD Bioscience). Total RNA was isolated using RNeasy Micro Kit Qiagen). For library construction mRNA was enriched from total RNA and submitted to fragmentation, cDNA synthesis, ligation of adapter and PCR amplification using the CATS RNA-seq Kit v2 x24 (Diagenode #C05010042) technology. Sequencing was performed using Nextseq500 (Illumina).

RNaseq was analyzed using classical tools. First raw files were aligned to mouse reference GRCm38/mm10 using STAR with quantMode option. Read mapping to multiple locations or too shorts were classically removed. The Bioconductor DESseq2 was then applied for count normalization and differential expression analysis between samples. Gene set enrichment analyses (GSEA) was

performed for the MSigDB hallmarks gene sets, using the Broad Institute software application (software.broadinstitute.org/gsea/download.jsp).

Quantitative reverse transcriptase-polymerase chain reaction (qRT-PCR)

RNA was isolated using RNeasy mini-Kit (Qiagen). Reverse transcriptase reaction (RT) was performed with 100 ng of total RNA using the Vilo mastermix superscript kit followed by polymerase chain reaction (qPCR) using PowerUp SYBR Green Master Mix on a StepOne Real Time PCR System (Thermoscientific). Expression levels were normalized by *hprt*. Primers sequences are available on demand.

DNA damage detection by immunofluorescence

Isolated cKIT positive cells were plated in Stempan medium (Stemcell) supplemented with cytokines, with or without MMC treatment (5ng/mL). Cells were fixed in 4% paraformaldehyde for 10 minutes at room temperature after 24 hours. Cells were subsequently plated in duplicate onto Poly-Prep slides (P0425-72EA, Sigma). Immunostaining was performed as described below, with each duplicate processed separately. Cells were permeabilized using 0.5% Triton X-100 in PBS for 10 minutes at room temperature and submitted to an initial incubation in TBS 1X saturation buffer with BSA 3% and Tween 0.1% for 1 hour. Incubation with primary antibodies: anti-53BP1 (ab21083, Abcam) or anti-γH2AX (05-636, Merck) was performed for 1.5 hours at room temperature. One hour Incubation was performed with secondary antibodies, either goat anti-mouse AF488 or goat anti-rabbit AF594 from Molecular Probes.A final staining with DAPI in mounting medium was applied before images acquisition on a AxioImager Z1 microscope with Axio Vision software (Zeiss). DNA damage foci were quantified using ImageJ.

Colony-Forming Unit

Lin$^-$ cells were spread in 1 mL cytokine-containing methylcellulose media M3434 (MethoCult medium, Stem Cell Technologies), and plated in 35 mm culture dish. Cells colonies were counted and replated after 7 days.

Engraftment.

Eight- to 12-week-old mice were used for cell isolation, and 8- to 10-week-old CD45.1 C57Bl/6-Boy/J wild-type mice were used as recipients for cell transplantation. BM cells were harvested from eight- to twelve-week-old wild-type Ly5.1 (CD45.1) and WT, WTTg, KO, KOTg (CD45.2) C57BL/6 mice. Cells were transplanted (1.5 × 10^6 test cells (45.2) and 0.2 × 10^6 competitor cells (45.1) into the tail vein of lethally irradiated (9 Gy) Ly5.1 (CD45.1) C57BL6/J mice. Cells were stained with APC anti-mouse CD45.2 (Clone A20), BV510 antimouse CD45.1 (Clone104) antibodies, all from BD Biosciences. The flow cytometry results were analyzed using FlowJo™ v10.9 Software (BD Life Sciences).

Ki-67 and Hoechst staining

Lin$^-$ cells were stained with antibodies directed against cell surface markers. After staining, cells were washed with PBS and then fixed with BD Cytofix/Cytoperm (BD Bioscience) for 20 min at 4 °C. After fixation, cells were washed twice with PermWash (BD Bioscience), re-suspended in 100 µl PermWash containing anti-Ki-67 and incubated a least 1hrs at 4 °C. Before analysis, bone marrow cells were incubated with Hoechst 33342 (1/1000 diluted) for 20 min at RT. The flow cytometry results were analyzed using FlowJo™ v10.9 Software (BD Life Sciences).

Annexin V staining

Lin$^-$ cells were stained with antibodies directed against cell surface markers. After staining, cells were washed with PBS. After harvest, cells were incubated for 15 min with annexin V according to the manufacturer’s instructions (Invitrogen) and then the flow cytometry results were analyzed using FlowJo™ v10.9 Software (BD Life Sciences).

Quantification and statistical analysis

All statistical analyses were performed by GraphPad Prism version 10. Student t-test or Wilcoxon-test were used for the comparisons of the means of two groups. Two-ways anova test was used for the comparisons of the means of more than two groups. The Log-rank test was used for the survival curve analyses. n represents biological replicates in cell culture experiments and the number of mice in animal studies.

Data and code availability

Sequencing data files have been deposited in NCBI’s Gene Expression Omnibus and are accessible under accession number GEO: GSE288125.

Supplemental Figures

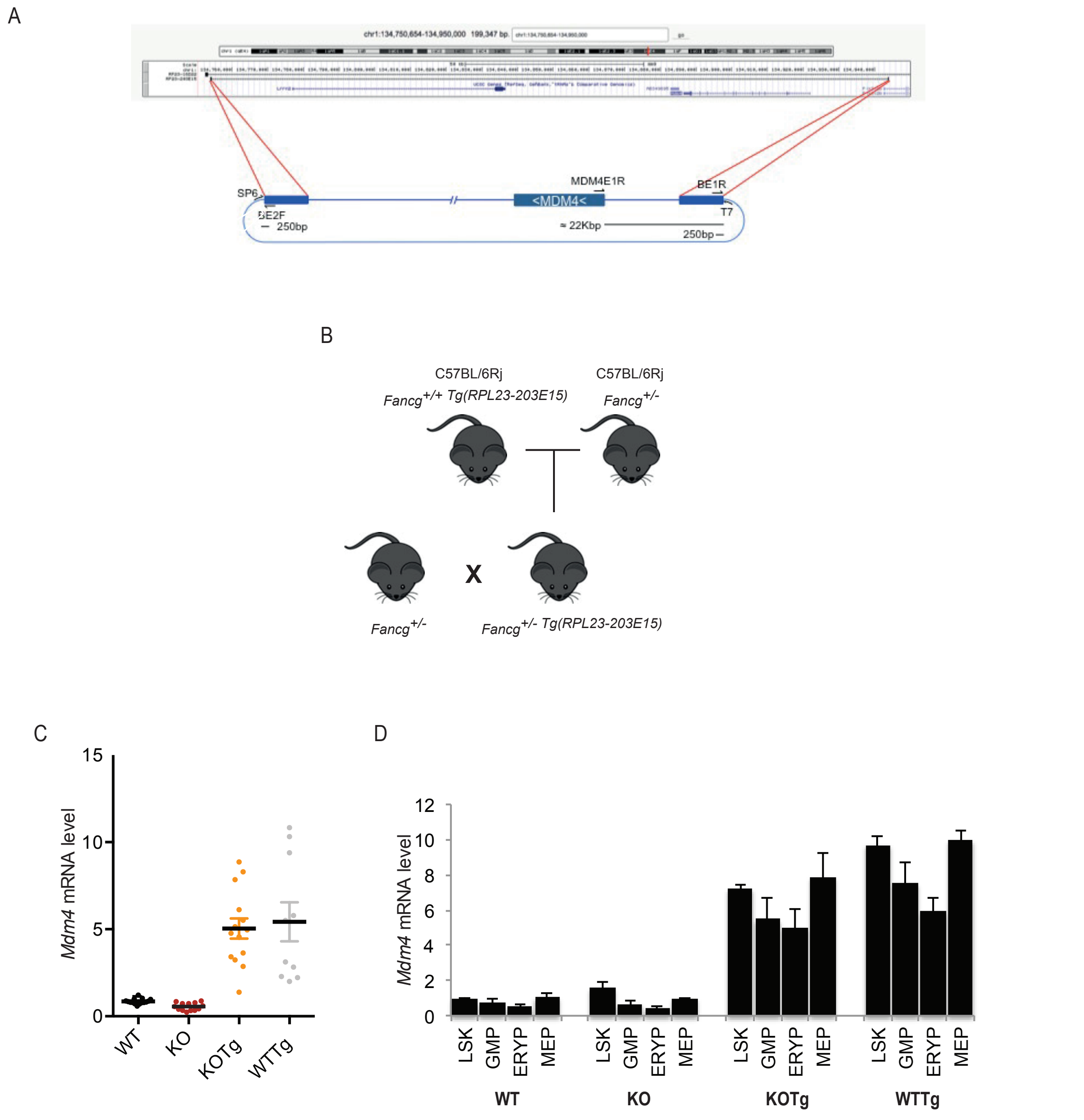

FigS1: (A) Schematic genomic region surrounding *Mdm4* and bacterial artificial chromosome (BAC; *RPL23-203E15*) that contains a wide genomic region surrounding *Mdm4* including its proximal regulatory sequences. (B) Crossed strategy to obtain all required genotypes: *Fancg*$^{-/-}$ (KO), *Fancg*$^{-/-\ Tg(RPL23\text{-}203515)}$ (KOTg), *Fancg*$^{+/+Tg(RPL23\text{-}203515)}$ (WTTg) and *Fancg*$^{+/+}$ (WT). (C) *Mdm4* mRNA level by Q-RT-PCR in PBMCs, (D) in LSK and hematopoietic progenitors of WT (n=3), KO (n=3), KOTg (n=3) and WTTg (n=3) mice.

FigS2

A

B

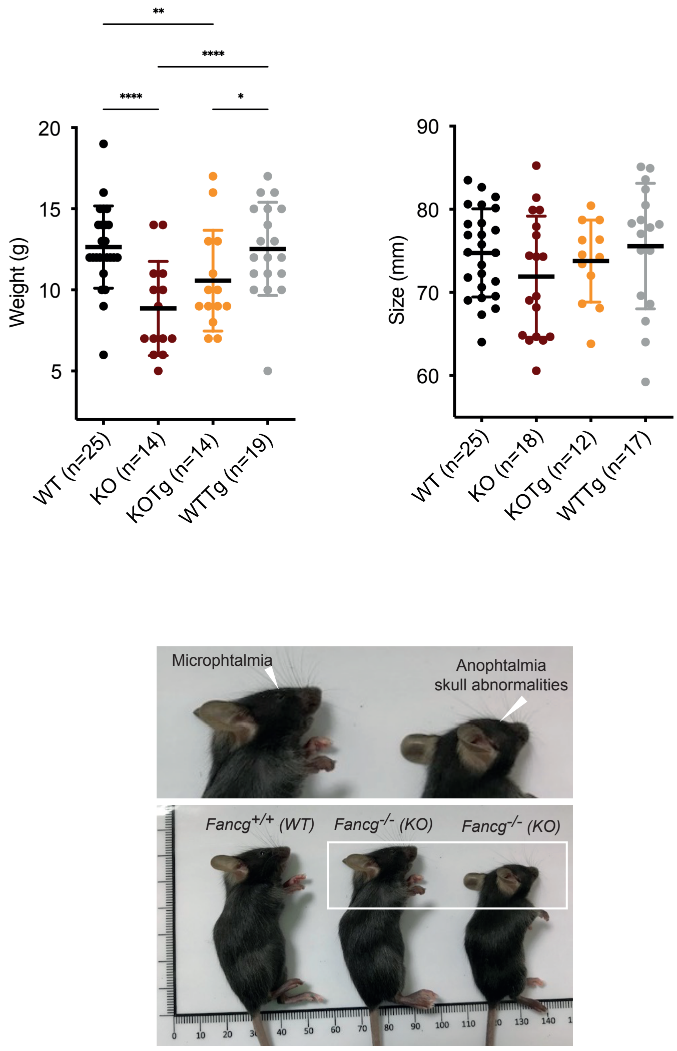

FigS2 (A) Weight and size from WT and KO mice 1-mo-old. (B) Representative picture of 3-mo-old WT and *KO* mice. The insert illustrates a magnified view of the growth defects.

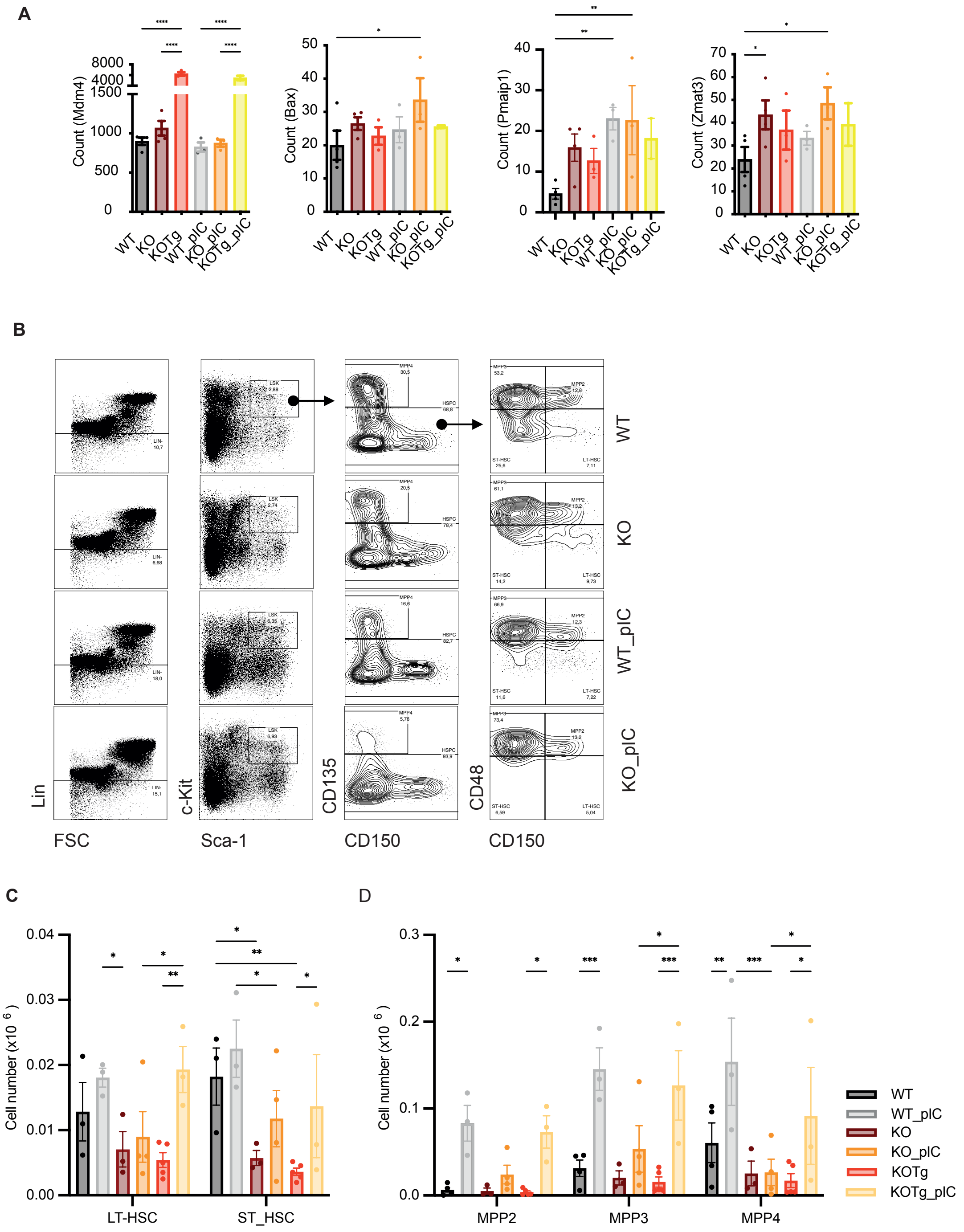

FigS3

(A) LSK mRNA levels of *Mdm4, Bax, Pmaip1 and Zmat3* from RNAseq data (count normalization) of KO (n=4), WT (n=4), KOTg (n=3), WT_pIC (n=3), KO_pIC (n=3), KOTg_pIC (n=2) LSK cells. Error bars represent mean ± SEM, two-ways ANOVA was used to determine p values (*$p < 0.05$). (B) Representative gating strategy used to identify and isolate LT-HSC, ST-HSC, MPP2, MPP3, and MPP4 based on expression of CD135, CD48, and CD150 in BM LSK. (C) Total number of BM LT-HSC ($Lin^-Sca\text{-}1^+c\text{-}Kit^+CD48^-CD150^+$), ST-HSC ($c\text{-}Kit^+Lin^-Sca\text{-}1^+$ $CD135^-$ $CD34^+$), or (D) multipotent progenitor (MPP2, $Lin^-Sca\text{-}1^+c\text{-}Kit^+$ $CD135^-$ $CD48^+$ $CD150^+$), (MPP3, $Lin^-Sca\text{-}1^+c\text{-}Kit^+$ $CD135^-$ $CD48^+$ $CD150^-$), (MPP4, $Lin^-Sca\text{-}1^+c\text{-}Kit^+$ $CD135^+$), Error bars represent mean ± SEM, two-ways ANOVA was used to determine p values (*$p < 0.05$).

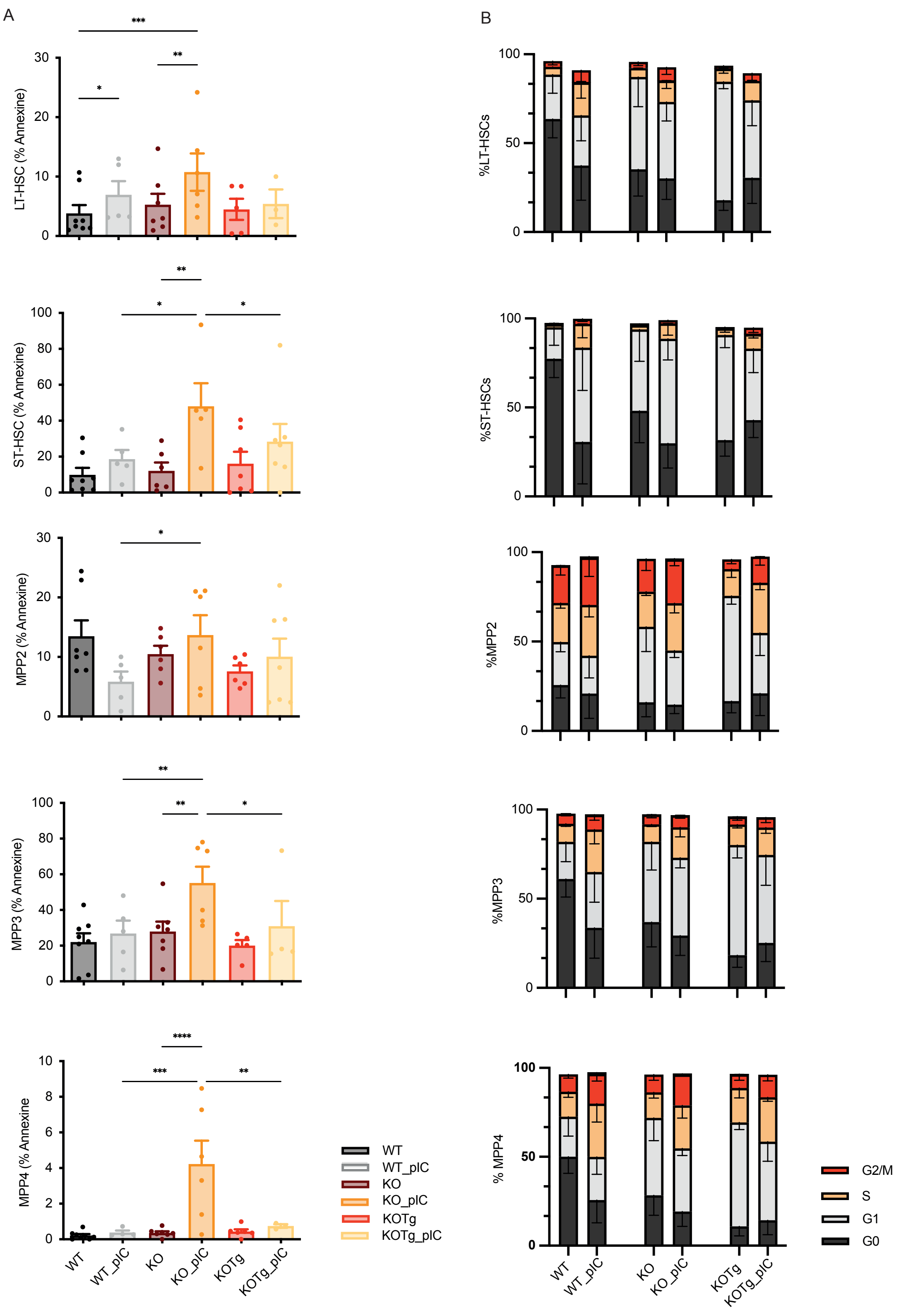

FigS4: Apoptosis and cell cycle analysis in LSK subset populations

(A) Apoptosis analysis of LSK cells in 8-12 weeks old WT (n=9), WT_pIC (n=8), KOTg (n=7), KOTg_pIC (n=3), KO (n=7) and KO_pIC *(n=5)* mice. Error bars represent mean ± SEM, two-ways ANOVA was used to determine p values (*p < 0.05). (B) Cell cycle analysis of LSK cells in 8-12-weeks old WT (n=4), WT_pIC (n=3), KOTg (n=3), KOTg_pIC (n=3), KO (n=4) and KO_pIC (n=4) mice (mean±SEM).

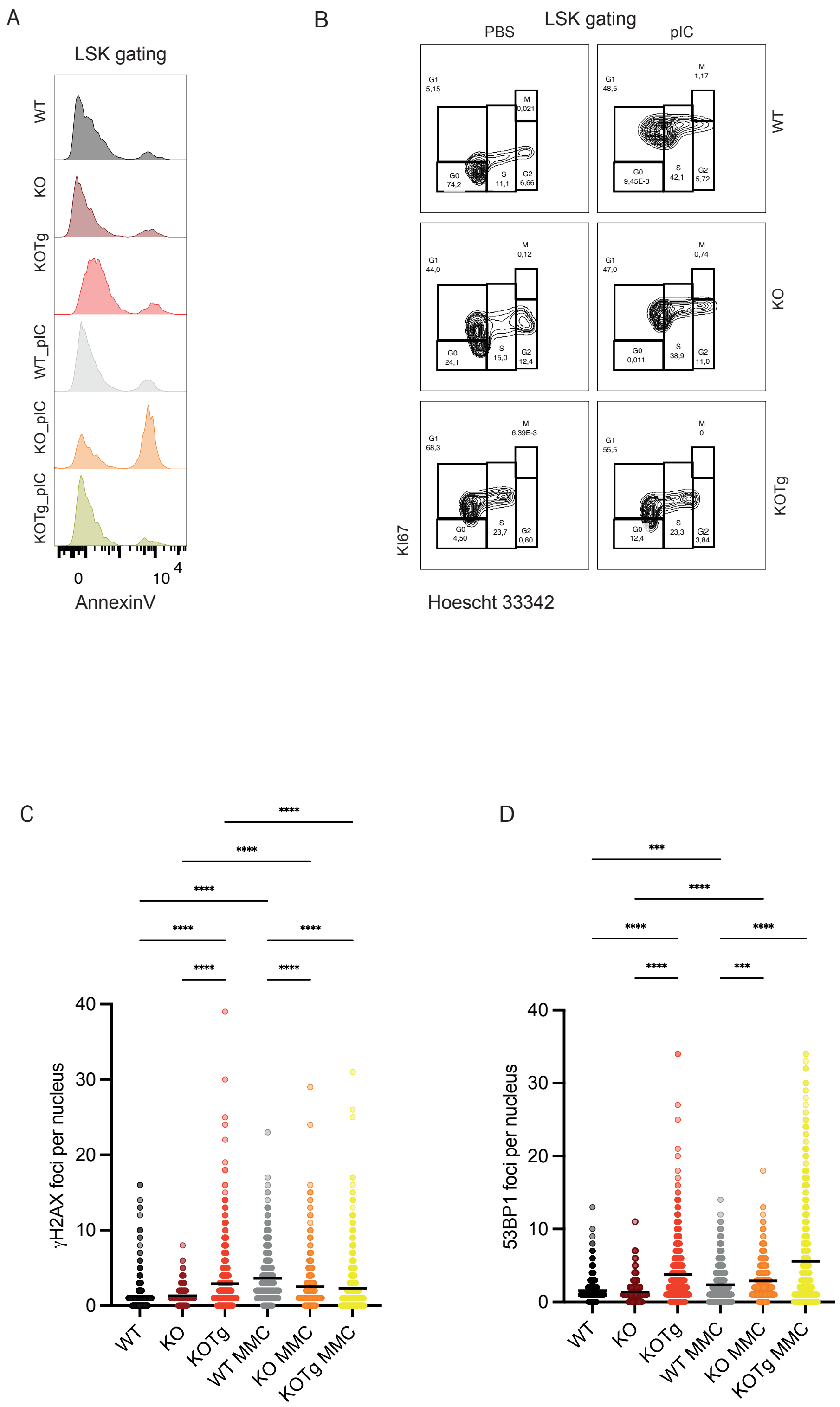

FigS5 Representative gating strategy on LSK cells used to identify apoptotic cells (A) and cell cycle stage. (B) Quantification analysis of γH2AX (C) and of 53BP1 (D) foci per cell. Errors bars shown mean+/- SEM. *p* values from Dunn's multiple comparisons test are shown. Shown is one representative experiment (n=1).
