# Supplementary material for: MDM4 overexpression alleviates developmental and hematopoietic defects in Fancg deficient mice
Source: Leukemia. 2025 Jul 25;39(10):2542–6. doi: 10.1038/s41375-025-02692-6 (PMC12463665; doi:10.1038/s41375-025-02692-6)
Supplement: Supplementary file 2 — Supplementary Table 1 [file 41375_2025_2692_MOESM2_ESM.pdf]

FCM antibodies panel

**Panel 1**	**Panel 2**	**Panel 3**
Pacific Blue™ Mouse Lineage Antibody Cocktail	Mouse Lineage Antibody Cocktail APC	Pacific Blue™ Mouse Lineage Antibody Cocktail
CD117 PercpCy5.5	CD117 PercpCy5.5	CD117 PercpCy5.5
Sca-1 PECy7	Sca-1 PECy7	Sca-1 PECy7
CD16/32 BUV737	CD48 APC-Cy7	CD16/32 BUV737
CD48 BV510	CD150 PE	CD48 BV510
CD150 BV650		CD150 BV650
CD105 AF647	*Post fixation*	CD105 AF647
CD41 BV605	Ki67 FITC	CD41 BV605
CD127 PE	Hoechst 33342	CD127 PE
CD135 PECy5		CD135 PECy5
		AnnexinV FITC
