# Supplementary material for: MDM4 overexpression alleviates developmental and hematopoietic defects in Fancg deficient mice
Source: Leukemia. 2025 Jul 25;39(10):2542–6. doi: 10.1038/s41375-025-02692-6 (PMC12463665; doi:10.1038/s41375-025-02692-6)
Supplement: Supplementary file 3 — Supplementary Table 2 [file 41375_2025_2692_MOESM3_ESM.pdf]

Primers

***Fancg* wt (400pb)**

Ex1-4F 5'GGCGACAATGTCCAGCCAGGTCATTCCAGC 3'

Ex1-4R 5'CTTGTAGAGTGAGGAGGAGTTCCCTAAGCC 3'

***Fancg* KO (227pb)**

HygF 5' GCATCATCGAAATTGCCGTCAACCAAGCTC 3'

HygR 5' TCGTGCACGCGGATTTCGGCTCCAACAATG 3'

***Mdm4* Tg**

Murine Mdm4 transgene genotyping #1 (252bp)

SP6 : ATTTAGGTGACACTATAG

BE2 : F GGTGGGTGCTAGGTAGGAGA

Murine Mdm4 transgene genotyping #2 (290bp)

BE1R : TTGTGGTTTCCCAGTCCCAG

T7 : TAATACGACTCACTATAGGG
